# Acute Lung Injury: From Molecular Circuits to System‐Level Therapeutics

**DOI:** 10.1002/mco2.70778

**Published:** 2026-05-26

**Authors:** Yaoli Hou, Sheng He, Lili He, Kun Liu, Wen Tang, Deming Wang, Jing Gui, Zhiying Zeng, Yan Wang, Wenjie Liu, Ren Jing

**Affiliations:** ^1^ Department of Medical Administration The Second Affiliated Hospital University of South China Hengyang Hunan China; ^2^ Department of Anesthesiology The Second Affiliated Hospital University of South China Hengyang Hunan China; ^3^ Department of Anesthesiology The First Affiliated Hospital University of South China Hengyang Hunan China; ^4^ Hengyang Medical School University of South China Hengyang Hunan China

**Keywords:** acute lung injury (ALI), endotypes, immunometabolism, network medicine, PANoptosis

## Abstract

Acute lung injury (ALI) and its severe manifestation, acute respiratory distress syndrome (ARDS), remain critical conditions with persistently high mortality. The failure to develop effective pharmacotherapies stems largely from reductionist approaches focused on isolated linear pathways. This review synthesizes recent breakthroughs redefining ALI as dysregulation of integrated pathological networks spanning immunity, metabolism, and cell death. We systematically analyze three interconnected core circuits: cGAS–STING as a central danger signal integrator, immunometabolic reprogramming as fuel for sustained inflammation, and the programmed cell death network—particularly PANoptosis—as executor of tissue damage. We further elucidate how ALI manifests as a multiorgan communication disorder, with the brain and gut actively shaping pulmonary inflammation. The convergence of single‐cell technologies, multiomics profiling, and computational modeling has deconstructed ARDS heterogeneity into clinically actionable endotypes (hyperinflammatory C1, hypoinflammatory C2) with differential treatment responses. This network‐based understanding is catalyzing a therapeutic shift toward rationally designed poly‐pharmacology, precision immunotherapies, and advanced platforms integrating smart nanomaterials with endogenous systems. By embracing this holistic perspective, we chart a course toward mechanism‐based, personalized interventions that move beyond supportive care to genuine disease modification.

## Graphical abstract

This visual summary illustrates the paradigm shift from linear pathways to network medicine in ALI. Three interconnected circuits—innate immunity, immunometabolism, and cell death (PANoptosis)—drive disease progression under multiorgan crosstalk (gut–lung, brain–lung). Multiomics deconstructs patient heterogeneity into endotypes (C1/C2), guiding network‐based therapeutics (nanomedicine, precision immunotherapy) toward personalized, homeostatic restoration. Created with BioRender.

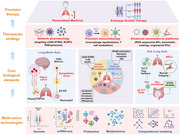

## 1 Introduction

Acute lung injury (ALI) and its severe manifestation, acute respiratory distress syndrome (ARDS), remain critical clinical syndrome characterized by diffuse alveolar damage, noncardiogenic pulmonary edema, and life‐threatening hypoxemia [1, 2, 3, 4]. Global mortality rate persists at 30–50%, establishing ALI/ARDS as a leading cause of death in intensive care units worldwide [5, 6, 7]. The LUNG‐SAFE study reported that 10.4% of intensive care unit (ICU) patients and 23.4% of mechanically ventilated patients met Berlin criteria, with overall mortality of 40% (34.9, 40.3, and 46.1% for mild, moderate, and severe ARDS, respectively) [8]. Notably, non‐Coronavirus Disease (COVID) ARDS mortality has remained static at 30–35% (mild) and 45–50% (severe) despite management advances [3, 8, 9, 10, 11]—a finding contested by studies reporting a modest overall decline over recent decades [9, 12, 13]. This discrepancy likely reflects differences in study populations, supportive care standards, and definitions, highlighting the need for standardized, context‐specific epidemiological surveillance.

Epidemiological estimates vary substantially by population. In 384,032 ventilated trauma patients, ARDS incidence fell sevenfold but mortality doubled (odds ratio [OR] 1.32) [9]; age modifies outcomes, with driving pressure (threshold 11 cm H_2_O) predicting mortality only in patients ≥80 years [14]. This finding challenges the one‐size‐fits‐all ventilation approach and advocates for age‐stratified mechanical ventilation. The perioperative period represents another major context, with over 312 million annual major surgeries exposing patients to iatrogenic insults—mechanical ventilation, ischemia–reperfusion, and transfusion—that trigger inflammation, edema, and barrier disruption [15, 16]. Postoperative pulmonary complications drive mortality, prolong hospitalization, and increase costs [17, 18, 19]. Yet despite advances in lung‐protective ventilation and conservative fluid management, no mechanism‐based pharmacotherapy exists, underscoring a decades‐long translational gap [20].

The profound heterogeneity of ALI/ARDS—in etiology (sepsis, pneumonia, aspiration, surgical trauma), clinical course, and treatment response—indicates pathophysiology far more complex than linear pathways can explain [20, 21]. This therapeutic impasse necessitates a paradigm shift. Rather than viewing ALI/ARDS as the product of isolated broken pathways, we propose that it represents the emergent phenotype of dysregulated molecular and cellular networks—a “network medicine” where immune dysregulation, metabolic reprogramming, and cell death pathways engage in bidirectional, self‐amplifying crosstalk. Evidence for systemic, network‐based dysfunction is accumulating: neural circuits (e.g., the corticotropin‐releasing hormone [CRH] neuron‐sympathetic nerve axis) centrally modulate lung inflammation [22]; the gut–lung axis, mediated by microbial metabolites, remotely controls alveolar immunity [23, 24, 25, 26]; and vascular signaling networks govern endothelial barrier integrity [27, 28, 29]. Even mechanical ventilation induces complex biological responses integrated into this network [30, 31]. This mechanistic complexity provides the blueprint for reconceptualizing ALI as a disease of interconnected networks.

**FIGURE 1:**
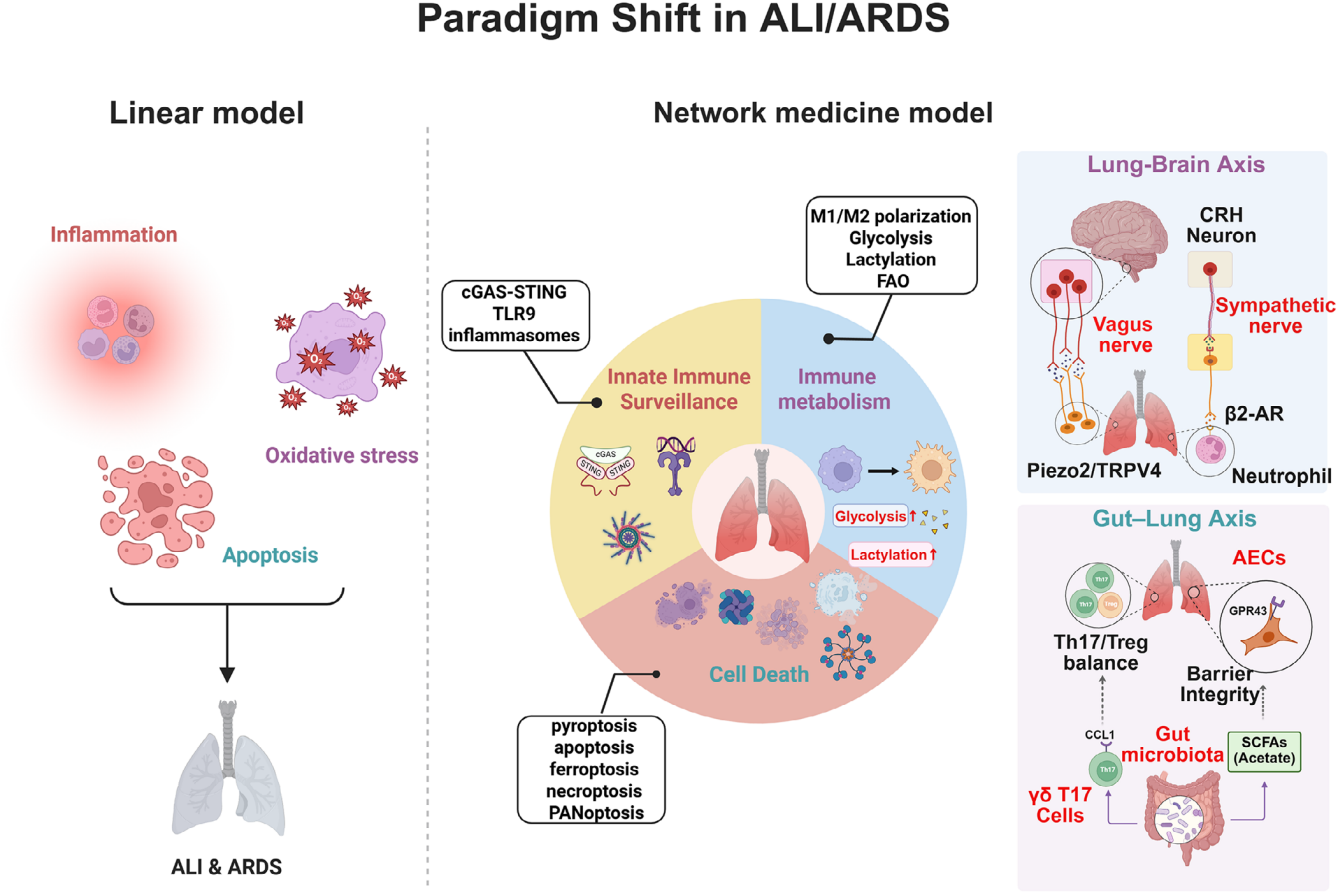
The paradigm shift in ALI/ARDS: from linear pathways to interconnected networks. Schematic representation of the conceptual evolution in ALI/ARDS pathogenesis. (A) Past linear model: isolated pathways of inflammation, oxidative stress, and apoptosis converge independently on lung injury. (B) Present network medicine model: the lung is a central hub within interconnected pathological circuits—innate immune surveillance (cGAS–STING, TLR9, inflammasomes), immunometabolism (M1/M2 polarization, glycolysis/FAO balance, lactylation), and cell death (pyroptosis, apoptosis, ferroptosis, necroptosis converging on PANoptosis). Dashed lines connect these circuits to multiorgan crosstalk (brain, gut). This framework explains clinical heterogeneity through maladaptive interactions within and between biological circuits. Figure created with BioRender (BioRender.com).

Aligned with this paradigm, this review has three objectives. First, to synthesize the core pathological circuits—dysregulated innate immunity, metabolic reprogramming, and regulated cell death—emphasizing their interdependencies. Second, to explore system‐level consequences, linking molecular circuit failures to tissue injury and organ dysfunction. Third, to critically assess emerging network‐informed therapeutics designed to target disease complexity rather than isolated components. Figure 1 contrasts the traditional reductionist view with the interconnected pathological circuits orchestrating ALI/ARDS.

## 2 The Core Pathological Network in ALI

ALI pathogenesis is driven by three interconnected molecular circuits that operate in parallel and engage in extensive crosstalk. This section deconstructs each circuit, highlighting their core components, regulatory nodes, and therapeutic vulnerabilities. The integration of these circuits into a unified pathological network is summarized in Table 1.

**TABLE 1:** Core pathological networks in ALI: mechanisms, interconnections, and therapeutic nodes.

Pathological circuit	Core components	Key interconnections	Pathological output	Druggable nodes (intervention level)
Innate immune surveillance	cGAS–STING, TLR9, NLRP3 inflammasome, AIM2, ZBP1	mtDNA activates both cGAS–STING and NLRP3 [29]; STING signaling primes NLRP3; cGAS–STING interacts with ZBP1 to promote PANoptosis [81, 82, 83]	Sustained hyperinflammation, cytokine storm, activation of multiple cell death pathways	Ligand: cfDNA/mtDNA scavenging (cationic nanoparticles) [31]; Sensor: cGAS/STING inhibitors (C176, TETAG–siRNA) [34, 35]; Upstream: Mitophagy enhancers (TAT–PBX1, IRGM) [30, 32]; Adaptor: ZBP1 inhibitors [83, 86, 87, 88]
Immunometabolic reprogramming	Glycolysis (HIF‐1α, PKM2, HK2, PFKFB2), FAO (CPT1A), lactylation, succinylation	Lactate drives lactylation (H3K18, EGR1, LPCAT2) promoting glycocalyx degradation and ferroptosis [49, 50, 51]; succinylation (STAT1 via CCL7–CCR1) drives M1 polarization [43]; glycolytic enzymes (GAPDH, ENO1) are central interaction hubs [116].	M1/M2 imbalance, barrier disruption, endothelial dysfunction, metabolic dysregulation	Enzymes: PKM2, HK2, PDK4 inhibitors; CPT1A activators [41, 46]; Metabolites: Lactate scavengers; SCFAs (acetate) supplementation [96, 97]; PTMs: Targeting lactylation or succinylation pathways; Transcriptional: HIF‐1α inhibitors [38]
Organelle stress and cell death	Mitochondria (mtDNA, mROS, mitophagy), ER (UPR, CIRP), PANoptosome (ZBP1, RIPK1, NLRP3, caspase‐8)	Damaged mitochondria release mtDNA (activates cGAS–STING) [29]; ER stress activates CIRP (activates ZBP1–PANoptosis) [84, 86]; impaired mitophagy (TRIM21‐mediated) exacerbates injury [71]; SQSTM1/p62 links autophagy to ferroptosis inhibition [78].	Alveolar–capillary barrier disruption, DAMP release, fibrosis, self‐amplifying cell death cycles	Organelles: Mitophagy inducers (IRGM, PINK1/Parkin activators) [30, 78]; Death platforms: ZBP1 inhibitors, PANoptosis blockers (Dachengqi decoction, Echinacea polyphenols) [82, 89, 90]; Antiferroptosis: GPX4 stabilizers, YAP/Nrf2 activators, SLC38A1 agonists [72, 73, 74, 75, 76, 77, 78, 79, 80]

This table synthesizes the three core pathological circuits that form the backbone of ALI pathogenesis. For each circuit, the key molecular components, their critical interconnections with other circuits, and the resultant pathological outputs are delineated. The “druggable nodes” column provides a hierarchical view of potential intervention points, ranging from ligand clearance to organelle quality control and direct pathway inhibition, highlighting the shift from targeting single effectors to modulating entire network hubs.

### 2.1 The Innate Immune Surveillance Circuit: cGAS–STING and Beyond as Signal Integrators

The cyclic GMP–AMP synthase (cGAS)–stimulator of interferon genes (STING) pathway has undergone a fundamental conceptual evolution within ALI/ARDS pathophysiology. No longer viewed as a single linear inflammatory cascade, it is now recognized as a central signaling hub and master integrator of a diverse danger signals. This pathway acts as a universal cytosolic DNA sensor, whose activation—whether triggered by pathogen‐derived DNA, endogenous mitochondrial DNA (mtDNA) released during cellular stress, or neutrophil extracellular trap (NET)‐derived cell‐free DNA (cfDNA)—establishes a common molecular trigger for sustained hyperinflammation [32, 33, 34].

The centrality of cGAS–STING is further cemented by its intricate crosstalk and positive feedback loops with parallel innate immune pathways, forming a robust self‐amplifying inflammatory network. Activation of this integrated network often originates from mitochondrial dysfunction, making mtDNA a universal pathogenic currency in ALI. In transfusion‐related ALI, recipient mtDNA acts as a potent “first hit” danger signal activating Toll‐like receptor 9 (TLR9) [35]. In sepsis‐induced ALI, autophagy deficiency (e.g., loss of autophagy related 16 Like 1 [ATG16L1]) in macrophages leads to mitochondrial reactive oxygen species (ROS) accumulation and mtDNA release, activating the cGAS–STING–NLR family pyrin domain containing 3 (NLRP3) axis and creating a pathogenic positive feedback loop that exacerbates lung injury [36]. Recent evidence demonstrates that immunity‐related GTPase M (IRGM) attenuates lipopolysaccharide (LPS)‐induced alveolar epithelial barrier dysfunction by inducing mitophagy and inactivating cGAS–STING signaling, positioning mitochondrial quality control as a fundamental upstream regulator of pulmonary inflammatory tone [37].

The translational promise of targeting this hub is highlighted by new therapeutic strategies moving beyond generic anticytokine approaches to directly modulate network core logic. These strategies can be categorized by intervention point: (1) ligand clearance using cationic nanoparticles to scavenge pathogenic cfDNA [38]; (2) mitochondrial stabilization through trans‐activator of transcription (TAT)–pre‐B‐cell leukemia homeobox 1 (PBX1)‐mediated enhancement of mitochondrial biogenesis [39] or compounds like glabridin that stabilize mitochondrial function and reduce mtDNA release [40]; (3) direct pathway inhibition employing small‐molecule STING inhibitors (e.g., C176) or advanced delivery systems like aminoguanidine‐assembled DNA tetrahedrons (TETAG) delivering STING small interfering RNA (siRNA) [41]; and (4) multitarget nanomedicine such as inhalable cobalt‐aluminumlayered double hydroxide (CoAl–LDH) nanosheets that combine ROS‐scavenging capability, direct DNA binding, and codelivery of STING inhibitors for synergistic network suppression at multiple nodes [42]. The intricate crosstalk between cGAS–STING and parallel DNA‐sensing pathways is systematically summarized in Table 2.

**TABLE 2:** Network of DNA‐sensing pathways in ALI/ARDS and their crosstalk with cGAS–STING.

Pathway	Primary sensor	Cellular localization	Key Activators (DAMPs/PAMPs) in ALI	Major downstream effects	Interaction with cGAS–STING axis	Therapeutic implications	Key references
cGAS–STING	cGAS	Cytosol	Cytosolic dsDNA (mtDNA, NET‐derived cfDNA, pathogen DNA)	Type I IFNs (IFN‐β), NF‐κB activation → TNF‐α, IL‐6, IL‐1β	*Core Hub*. Receives input from multiple DNA sources; output primes NLRP3 inflammasome	STING inhibitors (C176); cfDNA scavenging nanoparticles; TETAG–STING siRNA	[25, 26, 27, 31, 34, 35, 81]
TLR9	TLR9	Endosome/lysosome	Hypomethylated CpG DNA (extracellular mtDNA, bacterial DNA)	MyD88‐dependent NF‐κB activation → proinflammatory cytokines	*Parallel synergistic activation*. Extracellular mtDNA activates TLR9 as “first hit” alongside cGAS–STING [28].	TLR9 antagonists; mitochondrial stabilization to prevent mtDNA release	[28, 29, 33]
NLRP3 inflammasome	NLRP3	Cytosol	mtROS, K+ efflux, extracellular ATP, crystalline substances	Caspase‐1 activation → IL‐1β/IL‐18 maturation; GSDMD cleavage → pyroptosis	*Positive feedback loop*. cGAS–STING primes NLRP3 expression; pyroptosis releases more mtDNA, further activating cGAS–STING [29].	NLRP3 inhibitors; targeting upstream regulators (CD38, DDX3X)	[29, 65, 66, 67, 68, 69, 70, 71, 123, 132]
AIM2 inflammasome	AIM2	Cytosol	Cytosolic dsDNA (pathogen or host origin)	Caspase‐1 activation → IL‐1β/IL‐18 maturation; pyroptosis	*Shared ligand competition/cooperation*. Both bind cytosolic DNA; interactions may be synergistic or antagonistic.	AIM2 inhibitors (under investigation)	[27, 29]
ZBP1–PANoptosome	ZBP1	Cytosol	Z‐form nucleic acids; CIRP	Initiates PANoptosome assembly, converging pyroptosis, apoptosis, and necroptosis via RIPK1/RIPK3	*Convergent node*. Activated downstream of cGAS–STING or by mitochondrial stress, integrating DNA sensing into broader cell death programs	ZBP1 inhibition; PANoptosis blockers (Dachengqi decoction)	[82, 83, 86, 87, 88, 90]

This table delineates the key DNA‐sensing pathways implicated in ALI/ARDS pathogenesis. It highlights the central role of the cGAS–STING pathway as a signal integrator, its crosstalk with parallel and downstream effectors (TLR9, NLRP3, AIM2, and the PANoptosome), and the resulting amplification of inflammation and cell death. Therapeutic nodes at various levels of these pathways are indicated, with representative references.

### 2.2 The Immunometabolic Circuit: Directing Inflammation Through Cellular Reprogramming

#### 2.2.1 The Core Framework: Fueling Inflammation With Metabolic Reprogramming

The inflammatory response in ALI transcends linear cytokine release, constituting a dynamic process driven by profound cellular metabolic reprogramming [43, 44]. Under stress, immune cells—primarily macrophages and dendritic cells—and structural cells like alveolar epithelial and endothelial cells undergo rapid metabolic shift from oxidative phosphorylation toward glycolysis [45]. This “Warburg effect” provides essential biosynthetic precursors and regulates inflammatory signaling pathways, enabling specific functions such as proinflammatory macrophage polarization, dendritic cell maturation, and neutrophil recruitment [46, 47]. Notably, metabolic reprogramming acts as a double‐edged sword in ALI: while it fuels the initial inflammatory burst, excessive or prolonged glycolytic activation can drive pathological inflammation, and different immune cell subsets exhibit distinct metabolic features and functional consequences [45].

Emerging evidence reveals that metabolic interventions can confer protection against ALI. Aerobic exercise pretreatment significantly improves survival and attenuates histopathological damage in LPS‐induced ALI by restoring energy homeostasis, normalizing adenosine triphosphate (ATP)/adenosine diphosphate (ADP) and nicotinamide adenine dinucleotide (NAD+)/nicotinamide adenine dinucleotideNicotinamide adenine dinucleotide (NADH) ratios, and suppressing lactate accumulation through reprogramming linoleic acid and arachidonic acid metabolism [48]. Furthermore, trained immunity induced by β‐glucan attenuates ALI severity by upregulating glycolytic activity in alveolar macrophages via the AKT serine/threonine kinase 2 (AKT2)‐3‐phosphoinositide‐dependent protein kinase 1 (PDK1) axis, enabling these cells to sustain immune responses in high‐lactate environments [49].

#### 2.2.2 Cellular Metabolic Cross‐Talk: A Network Perspective

Metabolic reprogramming across different cell types forms a tightly interwoven, self‐amplifying pathological network through metabolite exchange and signal interactions [50]. Macrophage polarization state directly couples to metabolic phenotype [51, 52, 53]. Proinflammatory M1 macrophages highly depend on glycolysis, with nuclear translocation of pyruvate kinase M2 (PKM2) linked to ferroptosis during cytokine storms [43, 54]. Conversely, shift toward anti‐inflammatory M2 phenotype requires efficient mitochondrial fatty acid oxidation (FAO). The  carnitine palmitoyltransferase 1A (CPT1A)–interleukin (IL)‐10 axis drives this metabolic switch and promotes repair in ALI [48]. Microenvironmental signals regulate this process; endothelial‐derived Chemokine (C‐C motif) ligand 7 (CCL7) acts via C‐C chemokine receptor type 1 (CCR1) to induce signal transducer and activator of transcription 1 (STAT1) succinylation, licensing macrophages toward proinflammatory glycolytic M1 states [50].

As primary lung barrier components, metabolic stability of epithelial and endothelial cells is crucial for pulmonary homeostasis. In ALI, impairment of mitochondrial long‐chain FAO (mtLCFAO) in alveolar epithelial Type II cells actively regulates alveolar neutrophil infiltration by altering chemokine secretion (e.g., C‐X‐C motif chemokine ligand 2 [CXCL2]) [55]. In endothelial cells, downregulation of polyunsaturated fatty acid (PUFA) synthesis (e.g., reduced fatty acid desaturase [FADS1/2 expression) promotes ferroptosis and disrupts barrier integrity [56]. Mitochondrial dysfunction from defects in genes such as *Ndufb6* is a common upstream event contributing to injury in both epithelial and endothelial cells [44].

Lactate has evolved from metabolic end‐product to key signaling molecule. Lactate‐driven protein lactylation is widespread in ALI and exerts dual effects. Pathologically, phosphoinositide‐dependent protein kinase 4 (PDK4)‐driven lactate accumulation promotes lysophosphatidylcholine acyltransferase 2 (LPCAT2) lactylation in alveolar epithelial cells, suppressing solute carrier family 7 member 11 (SLC7A11) expression and triggering ferroptosis [57]. Concurrently, lactate induces lactylation of histone H3 at lysine 18 (H3K18) and transcription factor early growth response protein 1 (EGR1) (at K364) in endothelial cells, promoting glycocalyx degradation [58]. Protectively, in endothelial cells, H3K14 lactylation drives parkinsonism associated deglycase 7 (PARK7) upregulation, restoring protective FADS1/2‐dependent PUFA synthesis to counteract ferroptosis [56]. The cell type‐specific metabolic alterations fueling the inflammatory network in ALI are systematically summarized in Table 3.

**TABLE 3:** Metabolic reprogramming features and potential intervention strategies of key cell types in ALI.

Cell type	Primary metabolic alteration	Key regulatory molecules/pathways	Functional consequence	Potential therapeutic target	Key references
Alveolar macrophage (M1)	Marked enhancement of glycolysis (“Warburg effect”)	HIF‐1α, PKM2, FGF13–ERK axis, TREM‐1/mTOR/HIF‐1α pathway	Proinflammatory cytokine storm, aggravated tissue damage	Inhibit PKM2 nuclear translocation; inhibit HIF‐1α; target TREM‐1 signaling; PDK4 inhibitors	[36, 38, 39, 42, 44, 45, 47]
Alveolar macrophage (M2)	Enhanced mitochondrial fatty acid oxidation (FAO)	CPT1A–IL‐10 axis, PPAR‐γ	Resolution of inflammation, promotion of tissue repair	Activate CPT1A; PPAR‐γ agonists; deliver M2‐polarizing signals	[41, 46, 138, 139, 140]
Dendritic cell	Enhanced aerobic glycolysis	HIF‐1α–PFKFB2 axis	Promotes DC maturation, drives Th17 differentiation, activates adaptive immunity	Inhibit PFKFB2; DC‐specific HIF‐1α inhibitors	[39, 143]
Alveolar epithelial cell (Type II)	Impaired mitochondrial long‐chain FAO (mtLCFAO); increased glycolysis	Downregulated CPT1a; PDK4‐driven lactate accumulation	Barrier dysfunction, aberrant chemokine secretion (CXCL2), ferroptosis susceptibility	Enhance mitochondrial FAO (CPT1a agonists); PDK4 inhibitors; reduce lactate production	[45, 47, 48]
Pulmonary vascular endothelial cell	Enhanced glycolysis; reduced PUFA synthesis	FGF13–ERK/HIF‐1α axis; downregulated FADS1/2	Compromised barrier integrity, ferroptosis susceptibility, glycocalyx degradation	Inhibit FGF13; supplement Omega‐3 PUFAs; activate FADS1/2 (via PARK7)	[38, 46, 48, 49]
Neutrophil	Enhanced glycogenolysis and glycolysis	HIF‐1α; METTL3‐mediated SYK mRNA methylation	NET formation, ROS and proteolytic enzyme release, exacerbating tissue injury	Modulate glucose availability; inhibit glycolysis; target METTL3–SYK axis	[38, 144]

This table summarizes the cell type‐specific metabolic reprogramming that occurs during ALI. It details the primary metabolic shift, the key molecular regulators, the functional consequences for each cell type, and the resulting pathological outcomes. Potential therapeutic strategies aimed at correcting these metabolic imbalances are listed, along with supporting references. The table underscores the concept that metabolism is not a bystander but a central driver of immune and structural cell function in ALI.

#### 2.2.3 The Extended Network: Remote Organ Modulation via the Gut–Lung Axis

The pulmonary immunometabolic state is remotely governed by the gut microbiome via the gut–lung axis through metabolite signaling (e.g., short‐chain fatty acids[SCFAs], esculin), immune cell trafficking (γδ T17 cell migration), and barrier regulation [23, 25, 59, 60, 61, 62]. These mechanisms, spanning intracellular metabolic switches to posttranslational modifications, form a core immunometabolic circuit bridging initial injury to sustained inflammation [63, 64]. Accordingly, future therapies must target this network via small‐molecule metabolic modulators, metabolite scavenging, lung‐localized Clustered Regularly Interspaced Short Palindromic Repeats (CRISPR)/CRISPR‐associated protein 9 (Cas9) editing, or systemic microbiome reprogramming [59, 65].

### 2.3 The Organelle Stress and Cell Death Circuit: A Self‐Perpetuating Cycle of Damage

Beyond initial inflammation, ALI progression is propelled by a self‐amplifying circuit of organelle dysfunction and integrated programmed cell death (PCD). This circuit involves mitochondrial stress, endoplasmic reticulum (ER) stress, and multiple cell death modalities including pyroptosis, apoptosis, necroptosis, and ferroptosis, with extensive crosstalk creating a vicious cycle driving irreversible alveolar–capillary barrier disruption [66, 67]. Lung endothelial viability is essential for gas exchange; its inhibition represents a novel therapeutic strategy [68].

Pyroptosis, a highly inflammatory PCD form driven by inflammasome activation, is a central ALI driver across multiple cell types. Inflammasome activation stimulates pyroptosis initiation and subsequently releases inflammatory cytokines that drive ALI; this sustained pyroptotic inflammation, combined with shifts in macrophage polarization, creates a profibrotic microenvironment that promotes fibroblast activation and extracellular matrix deposition, mechanistically driving the transition from ARDS to pulmonary fibrosis [69, 70, 71].The canonical NLRP3 inflammasome pathway, culminating in gasdermin D (GSDMD) pore formation and IL‐1β/IL‐18 release, is a key therapeutic target [72]. Inhibition strategies are diverse: clinical‐stage GC‐1 ameliorates ALI by inhibiting macrophage NLRP3 assembly via the nuclear factor erythroid 2‐related factor 2 (Nrf2)‐p53‐apoptosis‐associated Speck‐like protein containing a CARD (ASC) axis [73, 74]; natural compounds like pelargonidin‐3‐O‐galactoside and ophiopogonin C target upstream regulators cluster of differentiation (CD) 38 and dead‐box helicase 3 X‐linked (DDX3X) respectively [75, 76]; and traditional formulations like Sanzi Yangqin Decoction exert protective effects by broadly inhibiting TLR2/ nuclear factor (NF)‐κB/NLRP3 signaling [77]. Apoptosis contributes significantly to alveolar epithelial and endothelial cell loss through ubiquitination‐mediated regulation; downregulation of E3 ligase tripartite motif‐containing protein (TRIM)21 leads to reduced degradation of oligoadenylate synthetase 3 (OAS3), promoting epithelial apoptosis via RNase L [78].

Ferroptosis has emerged as a pivotal mechanism characterized by iron‐dependent lipid peroxidation following glutathione depletion and glutathione peroxidase 4 (GPX4) inactivation [79, 80, 81]. Recent multiomics approaches have revealed that remimazolam alleviates LPS‐induced lung injury by inhibiting ferroptosis through upregulation of heme oxygenase‐1 (HO‐1) and restoration of the SLC7A11– glutathione (GSH)–GPX4 axis [82]. The protein arginine methyltransferase 1 (PRMT1)–EGR1– glutaminase 2 (GLS2) axis promotes ferroptosis by enhancing glutaminolysis [83]. Intercellular communication via macrophage‐derived extracellular vesicles carrying guanylate Binding Protein 2 (GBP2) promotes GPX4 degradation in endothelial cells [84]. Endogenous protective strategies include Yes‐associated protein (YAP)/Nrf2 axis transcriptional upregulation of GPX4 and SLC7A11 [85], celastrol‐mediated Hippo‐YAP pathway activation [86], and solute carrier family 38 member 1 (SLC38A1) promotion of autophagic degradation of iron transporter divalent Metal Transporter 1 (DMT1) [87].

**FIGURE 2:**
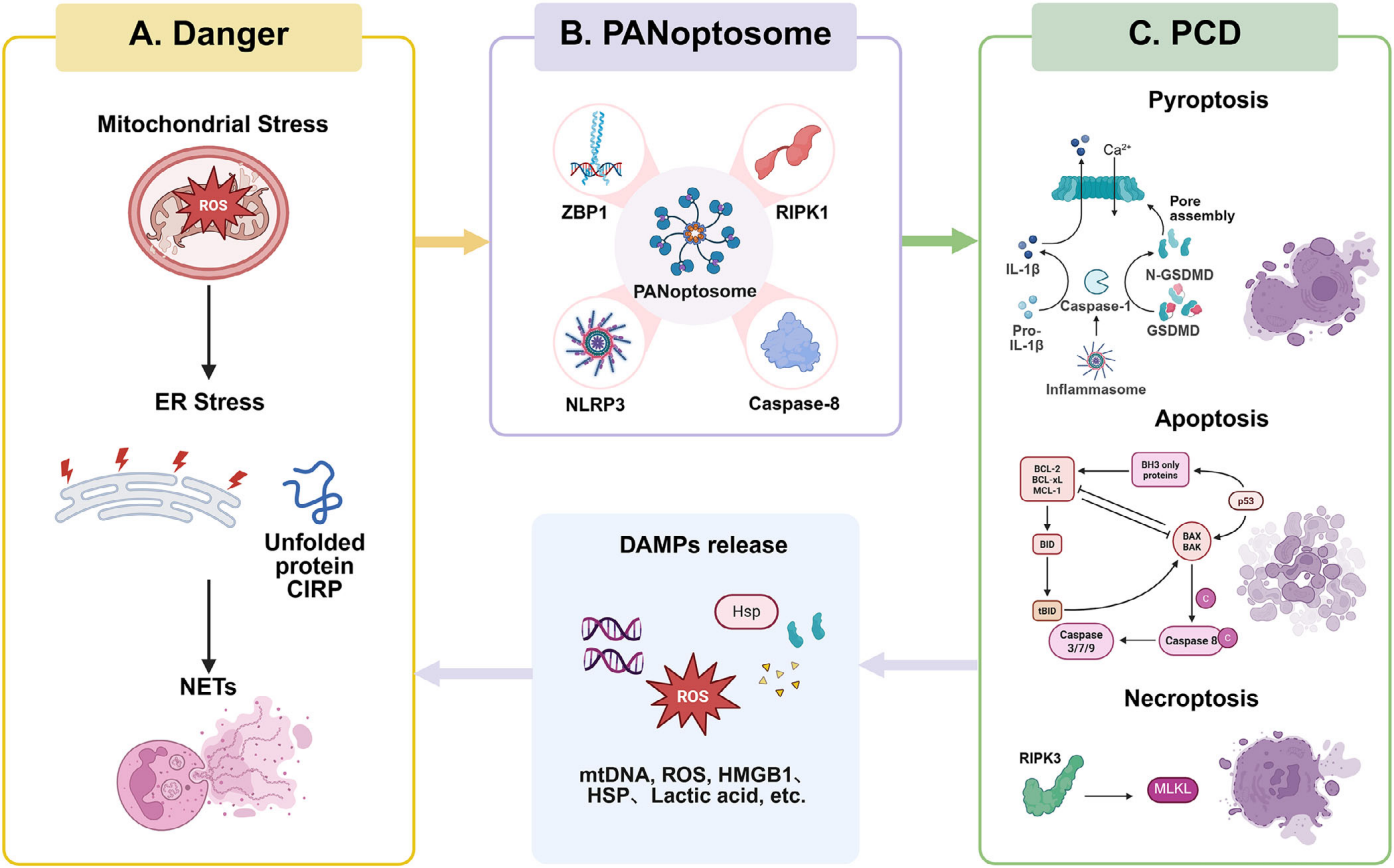
The integrated cell death circuit: the PANoptosis hub. Self‐amplifying cell death network centered on the PANoptosome complex (ZBP1, RIPK1, NLRP3, caspase‐8). Input signals (left) from mitochondrial DNA (mtDNA), ER stress (CIRP), and neutrophil extracellular traps (NETs) converge on the PANoptosome, activating three execution pathways (right): pyroptosis (NLRP3 → caspase‐1 → GSDMD → IL‐1β/IL‐18), apoptosis (caspase‐8 → caspase‐3), and necroptosis (RIPK3 → MLKL). A feedback loop (bottom) shows lytic cell death releasing DAMPs that exacerbate upstream organelle stress, creating a vicious cycle driving alveolar–capillary barrier disruption. This explains the superior efficacy of agents targeting shared nodes (e.g., ZBP1 inhibitors, PANoptosis blockers). Figure created with BioRender (BioRender.com).

Necroptosis, executed by the receptor‐interacting serine/threonine‐protein kinase (RIPK)1/RIPK3/mixed lineage kinase domain‐like protein (MLKL) cascade, can be triggered by NETs via the cGAS–STING pathway [88]. Critically, these major PCD pathways converge into PANoptosis—an integrated inflammatory death pathway governed by molecular platforms like the PANoptosome that plays a core role in lung injury [89, 90, 91, 92]. PANoptosis is initiated by innate immune sensors and driven by the PANoptosome complex, with Z‐DNA binding protein 1 (ZBP1) serving as a key sensor activated by stimuli like mtDNA or cold‐inducible RNA‐binding protein (CIRP) [90, 93, 94, 95]. This convergence explains the superior efficacy of agents targeting shared nodes, including Echinacea polyphenols inhibiting multiple death modalities simultaneously [96], and traditional formulas like Dachengqi decoction and Dachaihu decoction modulating upstream PANoptosis regulators [89, 97]. The self‐amplifying nature of this integrated cell death network is depicted in Figure 2, and the major PCD pathways are comprehensively summarized in Table 4.

**TABLE 4:** The programmed cell death network in ALI: mechanisms, regulation, and therapeutic targets.

Programmed cell death (PCD)	Core characteristics and key regulators	Primary pathogenic role and mechanism in ALI	Upstream triggers and downstream effects	Interaction with other death pathways	Representative therapeutic strategies/intervention targets	Key references
Pyroptosis	Inflammatory death; inflammasome (NLRP3) activation of caspase‐1 → GSDMD cleavage → pore formation, IL‐1β/IL‐18 release	Drives robust inflammation in macrophages, epithelial, endothelial cells; amplifies cytokine storm	Upstream: PAMPs/DAMPs (LPS, mtDNA, ATP, ROS), K+ efflux Downstream: Lytic death, cytokine/DAMP release	Core component of PANoptosis; cGAS–STING primes NLRP3; cross‐talk with apoptosis (caspase‐8)	Inflammasome assembly inhibitors (GC‐1) [66, 67]; CD38 ubiquitination promoters [68]; DDX3X–NLRP3 disruptors [69]; TLR2/NF‐κB/NLRP3 inhibitors [70]	[61, 62, 63, 64, 65, 66, 67, 68, 69, 70, 80, 89]
Apoptosis	Immunologically silent; caspase cascade (extrinsic: caspase‐8; intrinsic: caspase‐9) → effector caspase‐3	Contributes to alveolar epithelial and endothelial cell loss; disrupts barrier integrity	Upstream: Death receptors (extrinsic), mitochondrial/ER damage (intrinsic) Downstream: Cell shrinkage, nuclear fragmentation, apoptotic bodies	Cross‐talk with pyroptosis (caspase‐8 cleaves gasdermins) and necroptosis (caspase‐8 inhibits RIPK3)	Inhibit proapoptotic signaling (TRIM21–OAS3–RNase L axis) [71]; activate survival pathways (PI3K/AKT, cAMP/PKA)	[69, 71, 80, 89]
Ferroptosis	Iron‐dependent, nonapoptotic; lipid peroxidation from glutathione depletion and GPX4 inactivation	Causes lytic death of epithelial and endothelial cells; linked to oxidative stress and metabolic dysfunction; amplifies inflammation	Upstream: Iron overload, system Xc‐ inhibition, PUFA peroxidation, GPX4 defects Downstream: Membrane rupture, DAMP release	Interacts with autophagy (ferritinophagy), metabolism (glutaminolysis via PRMT1‐EGR1‐GLS2), and PANoptosis	Activate YAP/Nrf2 to upregulate GPX4/SLC7A11 [77]; inhibit PRMT1–EGR1–GLS2 [76]; SLC38A1 agonists [80]; ferrostatin‐1 [74]; multitarget inhibitors [72]	[70, 71, 72, 73, 74, 75, 76, 77, 78, 79, 80]
Necroptosis	Regulated necrosis; RIPK1/RIPK3/MLKL cascade → MLKL oligomerization, membrane rupture	Large‐scale cell lysis, DAMP release, potent secondary inflammation; key driver of severe injury	Upstream: Death receptors, TLRs, interferons, NETs via cGAS–STING [81] Downstream: Cell swelling, lysis, DAMP release	Converges with pyroptosis and apoptosis into PANoptosis; RIPK1 is key PANoptosome node	Lipid micelle‐encapsulated necroptosis inhibitors [135]; AMPK–RIPK1–MLKL modulation (spermidine) [136]; target upstream triggers (NETs/cGAS–STING) [81]	[77, 81, 135, 136]
PANoptosis	Integrated inflammatory death; PANoptosome platform (ZBP1, RIPK1, NLRP3, caspase‐8) engages pyroptosis, apoptosis, necroptosis components	Central driver of pathological tissue damage; creates self‐amplifying cycle of death and inflammation	Upstream: Innate immune sensors (ZBP1, AIM2) activated by DAMPs (mtDNA, CIRP) or PAMPs [82, 83, 84, 85, 86, 87, 88] Downstream: Simultaneous activation of multiple death effectors, massive inflammation	Master coordinator of PCD crosstalk; ZBP1 integrates signals from various pathways	Inhibit ZBP1 [83, 86]; modulate PI3K/AKT/NF‐κB (Dachengqi, Dachaihu decoctions) [82, 90]; suppress nitric oxide (Echinacea polyphenols) [89]	[79, 80, 81, 82, 83, 84, 85, 86, 87, 88, 89, 90]
Organelle dysfunction (upstream trigger)	Mitochondrial stress (mtDNA release, mROS, impaired mitophagy); ER stress (UPR, CIRP)	Unifying upstream trigger for multiple PCD pathways by generating DAMPs (mtDNA, CIRP) and disrupting homeostasis	Mitochondria: Damage → mtDNA release → cGAS–STING activation. ER: Stress → CIRP release → ZBP1–PANoptosis activation	Directly feeds into all PCD pathways; impaired quality control (mitophagy, ER‐phagy) exacerbates injury	Enhance mitophagy (IRGM, PINK1/Parkin activators) [30]; enhance ER‐phagy (FAM134B‐mediated by dendrobine) [78]	[29, 30, 78, 84]

This comprehensive table details the major programmed cell death (PCD) pathways involved in ALI/ARDS pathogenesis. It outlines their core characteristics, key regulators, and their specific roles in driving lung injury. Crucially, it highlights the extensive crosstalk between these pathways, particularly their convergence into the integrated PANoptosis platform. The upstream role of organelle stress (mitochondrial and ER) is also defined. For each pathway, representative therapeutic strategies that have shown efficacy in preclinical models are listed, demonstrating the shift from targeting single pathways to disrupting the broader cell death network. Key supporting references are provided for each entry.

While these three circuits are discussed separately, they do not operate in isolation. Emerging evidence suggests a temporal hierarchy: mitochondrial dysfunction often serves as an initiating event, with mtDNA release activating cGAS–STING early in the inflammatory cascade. This is followed by metabolic reprogramming that sustains inflammation through glycolytic activation and lactate‐driven modifications, ultimately culminating in cell death execution that amplifies the cycle through DAMP release. This hierarchical model, visually represented in Figure 1B, provides a framework for understanding how these circuits interact dynamically over the course of disease progression.

## 3 From Circuits to Systems: Multiorgan Crosstalk and Omics Insights

The molecular circuits described above do not operate in isolation; they are profoundly influenced by signals from distant organs. This section expands the analysis from intracellular pathways to interorgan communication networks and systems‐level approaches that are transforming our understanding of ARDS heterogeneity.

### 3.1 ALI as a Multiorgan Communication Disorder

ALI pathogenesis extends beyond the lung, emerging as a systemic disorder driven by dysregulated interorgan communication. Central to this network are the gut–lung and brain–lung axes, which facilitate bidirectional crosstalk through neural, immune, and humoral pathways [98, 99, 100]. Beyond these well‐characterized axes, emerging evidence implicates the kidney–lung axis (involving uremic toxins and fluid/electrolyte cross‐talk) and the bone marrow‐lung axis (involving emergency hematopoiesis and trained immunity) in ALI pathogenesis [98, 99, 101].

The gut–lung axis operates through defined cellular and molecular mechanisms. During sepsis, gut‐resident memory γδ T17 cells migrate to the lungs in a CCL1‐dependent manner, driving IL‐17A‐mediated pulmonary inflammation [25]. Lung tissue‐derived extracellular vesicles from septic animals carry miR‐128‐3p, which promotes M1 macrophage polarization and tumor necrosis factor (TNF)‐α/IL‐6 production by targeting Rab20 [102]. Microbial metabolites serve as critical messengers; SCFAs such as acetate, depleted in ALI models, preserve airway epithelial barrier integrity via G protein‐coupled receptor 43 (GPR43)–AMP‐activated protein kinase (AMPK) signaling [103, 104]. Compounds like forsythiaside A exhibit tissue‐specific actions, enhancing peroxisome proliferator‑activated receptor (PPAR)‐γ/retinoid X receptor (RXR)‐α in lung while suppressing TLR4/mitogen‐activated protein kinase (MAPK)/NF‐κB and myosin light chain kinase (MLCK)/myosin light chain 2 (MLC2) pathways in colon [23]. Polysaccharides from Houttuynia cordata exemplify gut‐initiated pulmonary protection by enriching Phocaeicola vulgatus and activating G Protein‐Coupled Receptor 43 (GPR43)‐mediated immunoregulation [105, 106].

**FIGURE 3:**
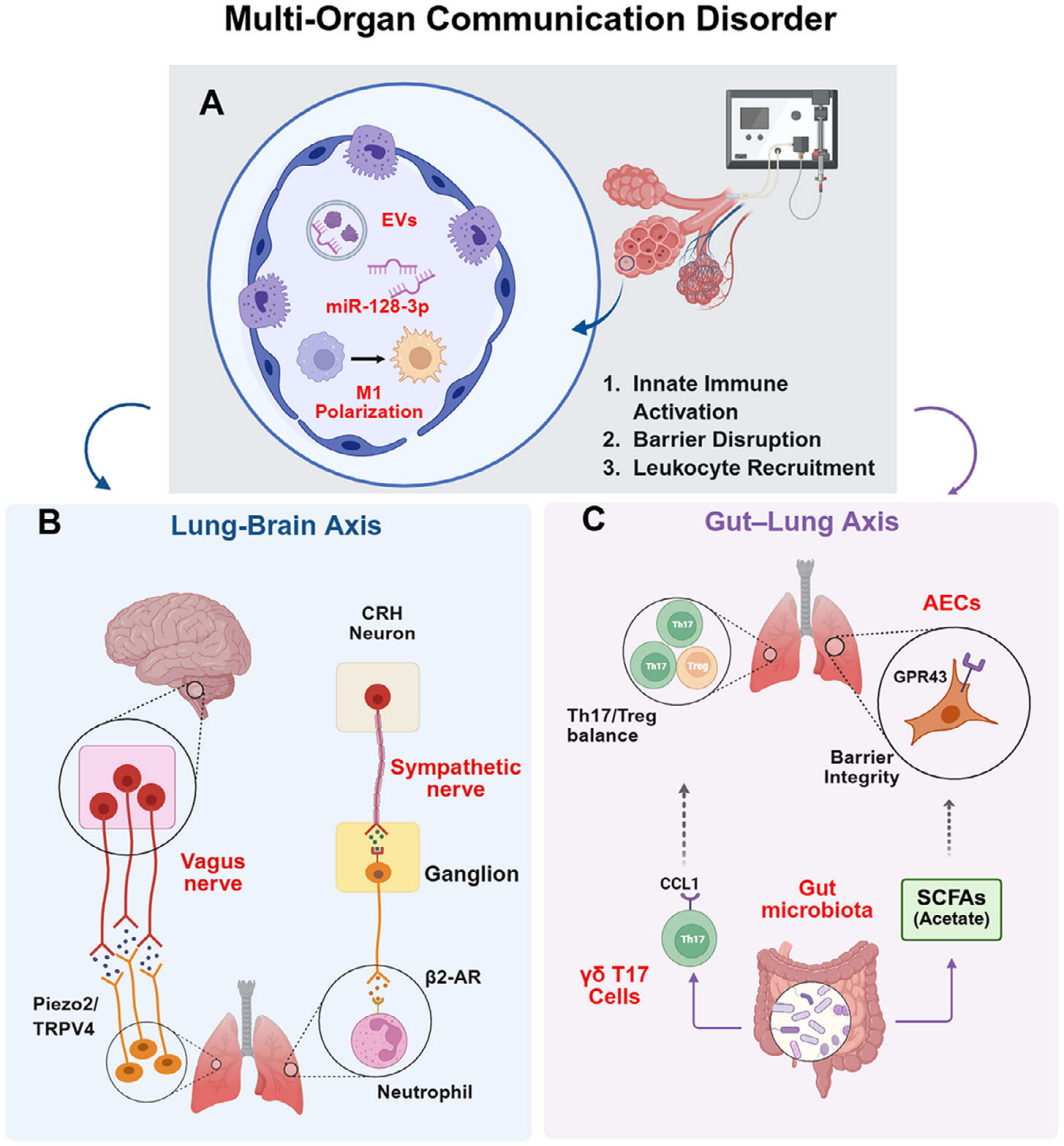
Multiorgan axes in ALI: the gut–lung and brain–lung dialogue. Bidirectional communication pathways defining ALI as a systemic disorder. Brain–lung axis: descending CRH neuron‐sympathetic output inhibits lung neutrophil NF‐κB via β2‐AR–β‐arrestin2; ascending mechanosensitive channels (Piezo2, TRPV4) triggering vagal neuroinflammation. The “triple‐hit” hypothesis integrates gut dysbiosis following brain injury as a third hit exacerbating lung injury. Gut–lung axis: γδ T17 cell migration drives IL‐17A‐mediated inflammation; microbial short‐chain fatty acids (acetate) preserve barrier integrity via GPR43–AMPK; extracellular vesicles carrying miR‐128‐3p promote M1 polarization. These axes converge on exaggerated innate immune activation, barrier disruption, and dysregulated leukocyte recruitment. Figure created with BioRender (BioRender.com).

The brain–lung axis operates through distinct but interconnected pathways. Following acute brain injury, CRH neurons in the hypothalamic paraventricular nucleus activate sympathetic output, releasing norepinephrine that signals lung neutrophils via beta‐2 adrenergic receptor (β2‐AR)–β‐arrestin2 to inhibit NF‐κB [22]. Mechanical ventilation conversely triggers ascending signaling: activation of lung mechanosensitive channels (Piezo‐type mechanosensitive ion channel component 2 [Piezo2], transient receptor potential cation channel subfamily V member 4 [TRPV4]) stimulates vagus nerve‐mediated neuroinflammation [107, 108, 109, 110]. The “triple‐hit” hypothesis further integrates these pathways, proposing that gut dysbiosis following brain injury constitutes a “third hit” exacerbating lung injury beyond sympathetic hyperactivity and primary insults [111, 112]. IL‐1‐driven emergency myelopoiesis in cranial bone marrow generates proinflammatory monocytes that infiltrate both brain and lungs [101], while glymphatic dysfunction releases damage‐associated molecular patterns (DAMPs) into systemic circulation, assaulting pulmonary endothelium [111]. Stroke‐induced upregulation of pulmonary angiotensin‐converting enzyme 2 (ACE2) further primes the lung for secondary infections [112]. Multitarget interventions such as Tongfu Xingshen capsule demonstrate therapeutic promise by simultaneously modulating brain, lung, and gut through microbiota and metabolic pathways [113]. The bidirectional communication pathways between the lung, gut, and brain are schematically summarized in Figure 3.

### 3.2 Systems Biology Vistas: Insights From Multiomics and Computational Models

The recognition that ARDS comprises multiple biologically distinct subtypes has catalyzed a shift from reductionist investigations toward systems‐level analyses. High‐throughput omics technologies now enable simultaneous measurement of thousands of molecular features, transforming heterogeneous observational data into mechanistically defined endotypes [114].

#### 3.2.1 Single‐Cell Transcriptomics Resolves Cellular Heterogeneity

Single‐cell RNA sequencing (scRNA‐seq) has fundamentally redefined the cellular cartography of the injured lung. In murine sepsis‐induced ALI, scRNA‐seq profiling identified tri‐lineage candidate biomarkers: secretoglobin family 3A member 2 (SCGB3A2) in epithelial cells, A‐kinase anchoring protein 12 (AKAP12) in endothelial cells, and CCL4 in monocytes/macrophages [115]. Among these, CCL4+ Mo/Mφ subpopulations exhibited disease‐specific lung infiltration and a distinct proinflammatory phenotype, with formyl peptide receptor 1 (FPR1) upregulated in hyperinflammatory clusters. Pharmacological FPR1 inhibition selectively reduced pulmonary infiltration of CCL4+ monocyte (Mo)/macrophage (Mφ) and attenuated sepsis‐induced ALI [115].

In ARDS models, alveolar Type II epithelial cells bifurcate into fibrotic and reparative transcriptional states, with balance modulated by wingless‐type MMTV integration site family (Wnt) signaling [116]. Macrophage heterogeneity extends beyond the M1/M2 dichotomy. Alveolar macrophages (AM) secrete transforming growth factor (TGF)‐β, restraining proliferation and inflammatory activity of monocyte‐derived macrophages (MDM) via Wnt pathway activation; this AM–MDM regulatory axis is essential for timely lung repair []. Pathogenic macrophage subsets can be selectively targeted: peptidyl arginine deiminase 2 (PAD2) catalyzes citrullination of NF‐κB p65 specifically in inflammatory macrophages during Pseudomonas aeruginosa pneumonia. PAD2‐inhibitor‐conjugated gold nanoparticles achieve selective delivery to M1‐polarized alveolar macrophages with marked therapeutic efficacy [118]. Beyond immune cells, scRNA‐seq has uncovered progenitor populations including lymphocyte antigen 6 family member A (Ly6a)+ alveolar epithelial subsets and endothelial cells with regional susceptibility [119].

#### 3.2.2 Multiomics Integration Defines Reproducible ARDS Endotypes

Translation of cellular heterogeneity into patient‐level phenotypic diversity requires integration of orthogonal omic layers. In a multicenter prospective cohort of 1048 ARDS patients, latent class analysis of 12 serum proteins identified three inflammatory phenotypes [116, 120]. Phenotype C1 exhibited intense innate immune activation, cytokine amplification, and metabolic reprogramming, with highest 90‐day mortality. Phenotype C2 displayed immune suppression and enhanced tissue repair with most favorable outcomes. Critically, glucocorticoids and higher PEEP improved outcomes in C1 but increased mortality in C2, demonstrating the clinical utility of endotype‐driven medicine [116, 120].

Longitudinal multiomics in trauma patients identified distinct thromboinflammation endotypes wherein elevated proteasome activation, catabolism, and superoxide formation specifically predicted subsequent lung failure [121]. In influenza‐induced lung injury, proteomic analysis revealed profound matrisome remodeling coinciding with emergence of a conserved myofibroblast activation state expressing *Tnc, Spp1, Grem1*, and *Cthrc1* [122]. Integrated proteomics and metabolomics identified upregulated sphingolipid signaling as a key pathological hub, with mitogen‐activated protein kinase kinase 1 (MAP2K1) as the central interactor [123]. An 8‐protein panel (vascular cell adhesion molecule 1 [VCAM1], lactate dehydrogenase B [LDHB], moesin [MSN], filaggrin 2 [FLG2], transgelin 2 [TAGLN2], lamin A / lamin A/C [LMNA], mannose‐binding lectin 2 [MBL2], lipopolysaccharide‐binding protein [LBP]) was validated in an independent cohort, achieving superior prognostic accuracy [123].

Sphingolipid metabolism emerged as a critical pathological hub; obesity‐associated ARDS involves ceramide transfer protein dysregulation, driving ceramide accumulation and alveolar macrophage apoptosis [124]. Multiomics further identified tyrosine 3‐monooxygenase/tryptophan 5‐monooxygenase activation protein epsilon (YWHAE) as a central ferroptosis mediator, with glutathione and cysteine metabolism as key pathways [125]. Platycodon grandiflorum‐derived nanoparticles regulate macrophage inflammation via glycolysis and lipid metabolism [126]. Lactylation signatures correlate with alveolar immune microenvironment composition, defining a novel endotype [127, 128, 129]. Competing endogenous RNA networks orchestrate cell‐specific responses modulating epithelial apoptosis, endothelial permeability, and macrophage polarization [130]. The gut–lung axis constitutes an additional trans‐kingdom omic layer; metagenomic and metabolomic integration demonstrated that Kuqin enriches Akkermansia muciniphila, whose abundance inversely correlates with pulmonary indoleamine 2,3‐dioxygenase 1 (IDO1) activity [104]. Orally administered onion‐derived mitochondria transit intact from gut to lung, are internalized by macrophages, and deliver methyl 3,4‐dihydroxybenzoate, which epigenetically suppresses mt‐NADH dehydrogenase subunit 1 (ND1) expression and reduces oxidative stress  [131]. These convergent findings are systematically summarized in Table 5.

**TABLE 5:** Systems biology approaches in ARDS: from cellular heterogeneity to clinical endotypes.

Technology/approach	Key findings/utility	Identified biomarkers/cell states/endotypes	Clinical implications/therapeutic opportunities	Key references
Single‐cell transcriptomics	Resolved cellular heterogeneity; identified pathogenic subpopulations and progenitor states	Epithelial cells: SCGB3A2+; AT2 fibrotic (Igfbp6+, Gstm1+) vs. reparative (Tgm2+, Anxa1+); Ly6a+ progenitors [108, 110, 112] Endothelial cells: AKAP12+; Lrg1+, Ucp2+ capillary subsets [108, 112] Myeloid cells: CCL4+ FPR1+ Mo/Mφ; PAD2+ inflammatory macrophages [104, 106, 111] Fibroblasts: Tnc+, Spp1+, Grem1+, Cthrc1+ myofibroblasts [115]	Targeted therapy: PAD2 inhibitor‐nanoparticles for M1 macrophages [111]; FPR1 inhibition to block CCL4+ Mo/Mφ infiltration [104]	[104, 106, 108, 110, 111, 112, 115]
Multiomics integration (proteomics + transcriptomics)	Defined reproducible inflammatory endotypes with differential treatment responses	Three phenotypes (C1, C2, C3) from latent class analysis of 12 serum proteins (*n* = 1048): C1 hyperinflammatory (highest mortality), C2 hypoinflammatory (favorable), C3 intermediate [108, 113]	Precision medicine: Glucocorticoids/higher PEEP improve C1 outcomes but increase C2 mortality; 12‐biomarker classifier enables prospective endotype assignment [108, 113]	[108, 113]
Proteomics and metabolomics	Identified pathological hubs (sphingolipid metabolism, ferroptosis); validated prognostic panels	8‐protein panel: VCAM1, LDHB, MSN, FLG2, TAGLN2, LMNA, MBL2, LBP (AUC 0.802) [116] Sphingolipid metabolism: CERT dysregulation drives ceramide accumulation in obesity–ARDS [117] Ferroptosis: YWHAE as central mediator; glutathione/cysteine metabolism as key pathways [118]	Novel biomarkers for prognosis/stratification; therapeutic targeting of CERT, YWHAE, or glutathione metabolism	[116, 117, 118]
Computational models and network medicine	Real‐time VILI prediction; identified druggable network modules	Mechanical model: CFVent area from first 15 min ventilation predicts VILI outcomes at 4 h [127, 128] Machine learning: Four‐gene signature (DDAH2, PNPLA2, STXBP2, TCN1) with high diagnostic performance [125, 126] Network modules: GBP2–OTUD5–GPX4 axis as ferroptosis driver; plantainoside D as inhibitor [77]	Real‐time personalized ventilator management; network‐based drug targets (e.g., plantainoside D for GBP2–OTUD5)	[77, 125, 126, 127, 128]
Epigenomics and noncoding RNAs	Identified novel regulatory layers and endotypes	Lactylation signatures: Correlate with alveolar immune microenvironment, defining novel ARDS endotype [120, 121, 122] ceRNA networks: lncRNA–miRNA–mRNA axes modulate epithelial apoptosis, endothelial permeability, and macrophage polarization [123]	Epigenetic therapies; targeting specific ceRNA networks or lactylation pathways	[120, 121, 122, 123]

This table summarizes the major systems biology approaches that have been applied to deconstruct the heterogeneity of ARDS. It highlights the power of single‐cell technologies to resolve cellular heterogeneity, multiomics integration to define clinically actionable endotypes, and computational modeling to enable real‐time prediction and drug discovery. The identified biomarkers, cell states, and endotypes are listed alongside their clinical implications and potential therapeutic opportunities, with key supporting references.

#### 3.2.3 Computational Models Enable Biomarker Discovery and Clinical Prediction

High‐dimensional omics data demand sophisticated computational architectures. In sepsis‐induced ALI, ensemble machine learning applied to transcriptomic datasets converged on a four‑gene signature (dimethylarginine dimethylaminohydrolase 2 [DDAH2], patatin‐like phospholipase domain‐containing protein 2 [PNPLA2], syntaxin binding protein 2 [STXBP2], transcobalamin 1 [TCN1]) with excellent diagnostic performance across independent validation cohorts [132, 133]. Weighted gene coexpression network analysis identified the MEblue module, enriched for immune activation pathways, as most robustly associated with disease severity [132].

Mechanistic computational modeling offers a distinct approach. A compartment model of lung mechanics, parameterized by forced oscillation measurements, was developed to predict ventilator‐induced lung injury (VILI) [134, 135]. The model‐derived compliance factor outperformed driving pressure and mechanical power in forecasting subsequent injury severity. The continuous‐flow ventilation (CFVent) area calculated from the first 15 min of ventilation accurately predicted VILI outcomes 4 h later, representing a significant advance toward real‐time personalized ventilator management [135].

Network medicine frameworks integrate disparate data types into coherent disease maps. Projecting multiomic alterations onto protein–protein interaction networks enables identification of disease modules—localized, densely interconnected regions of the interactome perturbed in ARDS. The GBP2–OTU deubiquitinase 5 (OTUD5)–GPX4 axis, identified as a driver of endothelial ferroptosis, constitutes a compact druggable module; plantainoside D was validated as a potent small‐molecule inhibitor of this interaction [84].

#### 3.2.4 Convergence and Future Directions

**FIGURE 4:**
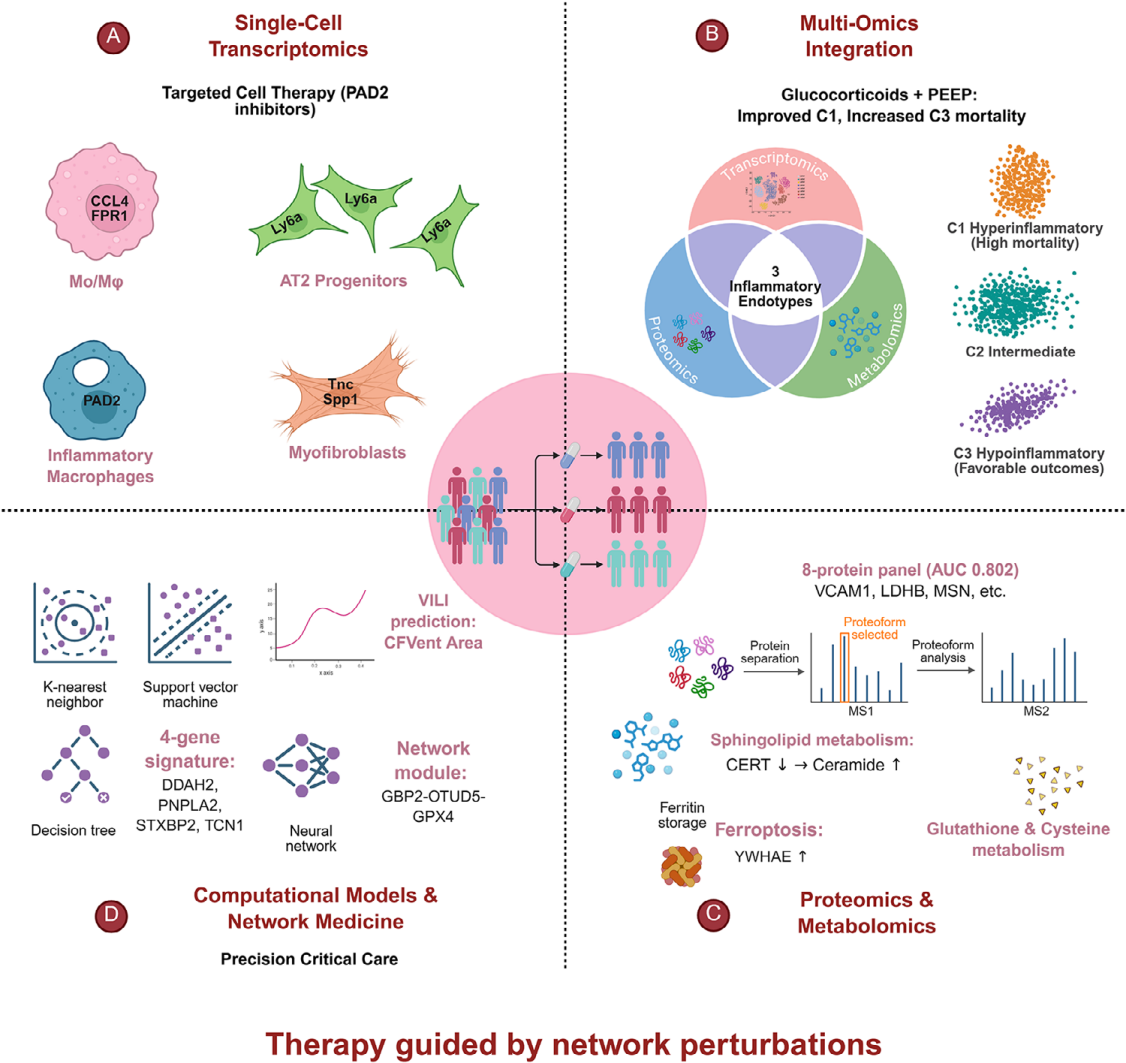
Systems biology deconstructs ARDS heterogeneity: from cellular states to clinical endotypes. Multiomics integration resolves ARDS heterogeneity into actionable endotypes. (A) Single‐cell transcriptomics identifies pathogenic subpopulations: CCL4+ FPR1+ monocytes/macrophages, Ly6a+ alveolar progenitors, PAD2+ inflammatory macrophages, and fibrotic myofibroblasts (Tnc+, Spp1+). (B) Multiomics integration defines three inflammatory endotypes (C1 hyperinflammatory, C2 hypoinflammatory, C3 intermediate) with differential responses to glucocorticoids/PEEP. (C) Proteomic/metabolomic profiling reveals sphingolipid metabolism (CERT dysregulation), ferroptosis (YWHAE), and an 8‐protein prognostic panel. (D) Computational modeling enables VILI prediction and identifies druggable modules (e.g., GBP2‐OTUD5‐GPX4). Figure created with BioRender (BioRender.com).

The convergence of multiomics technologies with advanced computational analytics is reconfiguring ARDS research. Heterogeneity that long confounded clinical trials is now systematically deconstructed into constituent molecular endotypes with distinct therapeutic vulnerabilities. Systems biology is moving ARDS toward a precision critical care paradigm wherein therapy is guided by specific network perturbations active in an individual patient. The integration of these approaches is comprehensively summarized in Figure 4.

## 4 Therapeutic Translation: Engineering Network Pharmacology

**FIGURE 5:**
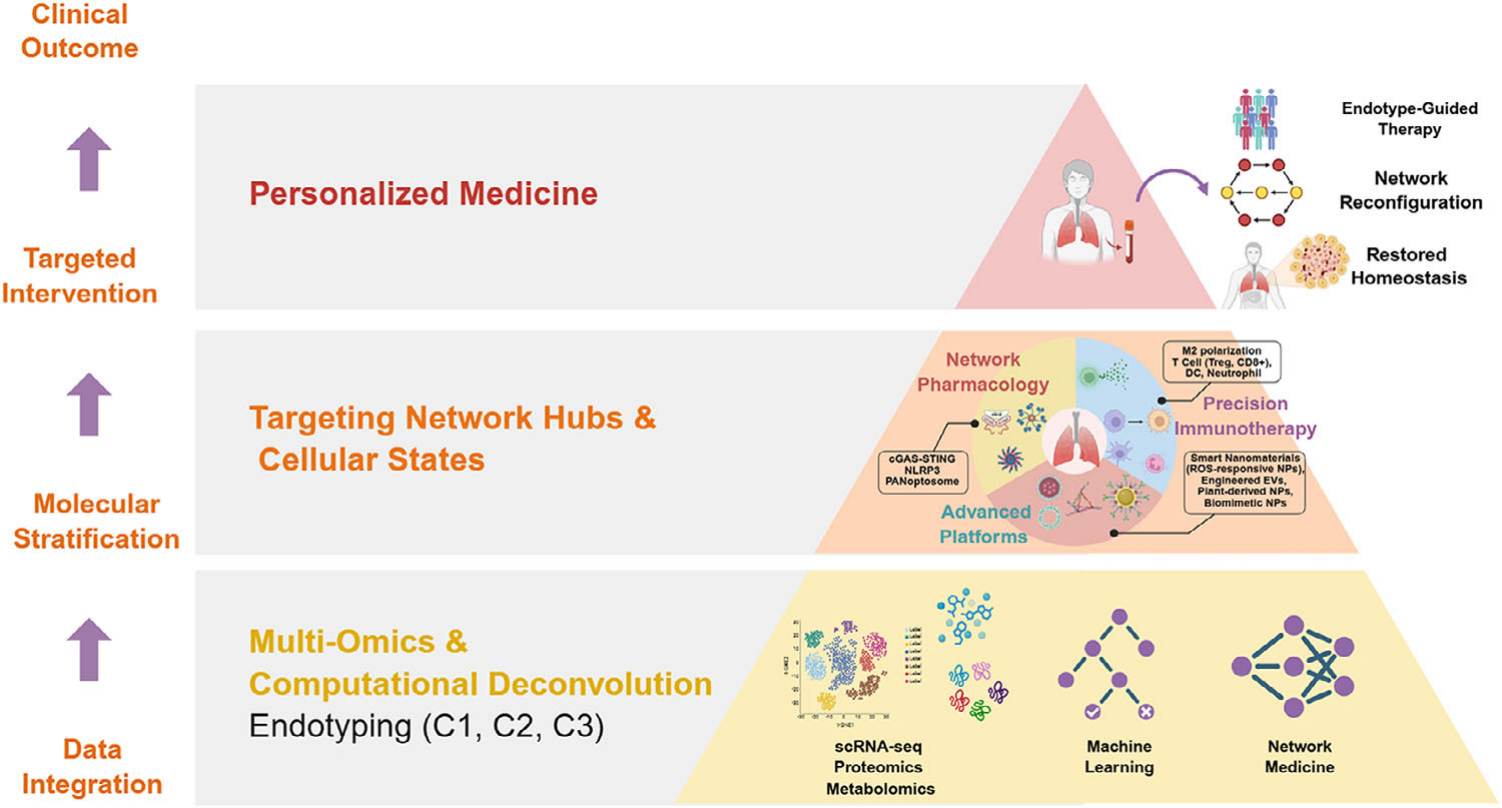
A roadmap for network‐based therapeutics in ALI. Hierarchical pyramid translating systems‐level understanding into personalized therapy. Base layer (foundation): multiomics and computational deconvolution deconstruct heterogeneity into endotypes (C1, C2, C3). Middle layer (strategy): three interconnected modules targeting network complexity—network pharmacology (cGAS–STING, NLRP3, PANoptosome); precision immunotherapy (macrophage repolarization, T cell modulation, DC targeting, neutrophil inhibition); advanced platforms (smart nanomaterials, engineered EVs, biomimetic NPs, plant‐derived vesicles). Top layer (goal): endotype‐guided personalized medicine achieving durable network reconfiguration and restored homeostasis. Figure created with BioRender (BioRender.com).

The systems‐level understanding of ALI as a network disorder necessitates a corresponding evolution in therapeutic strategy. Rather than targeting isolated effectors, next‐generation therapies are designed to disrupt network hubs, reprogram cellular states, and harness endogenous reparative mechanisms. The translation of systems‐level understanding into personalized therapeutic interventions follows a logical progression from molecular deconvolution to targeted strategies, as outlined in the hierarchical roadmap presented in Figure 5.

### 4.1 Network Pharmacology: Targeting Circuit Hubs and Modules

ALI is sustained by interdependent molecular circuits, not isolated effectors. Its network topology—defined by highly connected hubs (STING, NLRP3) and convergent modules (PANoptosis, macrophage–epithelium crosstalk)—renders single‐target strategies ineffective [136, 137].

cGAS–STING permits multilevel hub engagement. This pathway integrates mtDNA, NET‐derived DNA, and pathogen DNA into a common inflammatory output [88, 138]. Its nodal vulnerability permits intervention at three hierarchical levels: ligand clearance using cationic nanoparticles [38]; signal transducer inhibition with inhalable CoAl–LDH nanosheets delivering STING inhibitor C176 [42]; and upstream mitochondrial stabilization via TAT‐PBX1 [39] or glabridin [40].

NLRP3 inflammasome represents another high‐priority hub. GC‐1 inhibits inflammasome assembly via Nrf2–p53–ASC axis [73, 74]; pelargonidin‐3‐O‐galactoside promotes CD38 ubiquitination, elevating NAD+ and silent information regulator 1 (SIRT1) to suppress NF‐κB p65 acetylation [75]; ophiopogonin C directly disrupts the DDX3X–NLRP3 protein–protein interface [76]. Multiherb formulations such as Shuangdan Jiedu Decoction and the Lonicerae–Forsythiae pair simultaneously suppress both cGAS–STING and NLRP3, demonstrating combined hub inhibition achievable with rationally designed poly‐pharmacology [130, 139].

PANoptosis represents a coordinated death platform initiated by ZBP1 and RIPK1 [91, 96]. Dachengqi decoction and Dachaihu decoction inhibit PANoptosis by suppressing ZBP1/RIPK1 expression and modulating phosphoinositide 3‐kinase (PI3K)/AKT/NF‐κB signaling [89, 97]. Echinacea polyphenols concurrently inhibit all three death modalities through nitric oxide suppression [140]. Beyond molecular hubs, discrete cellular modules are tractable: Sanzi Yangqin Decoction targets CD40 on alveolar macrophages to inhibit epithelial necroptosis [141]; lipid micelle‐encapsulated necroptosis inhibitors achieve cell‐type‐selective MLKL targeting [142]; spermidine attenuates necroptosis via AMPK–RIPK1–MLKL signaling [143]. The therapeutic frontier lies in rationally designed poly‐pharmacology that simultaneously engages multiple vulnerable nodes within the cGAS–STING–NLRP3–PANoptosis axis [88, 91, 137].

### 4.2 Precision Immunotherapy: Targeting Cellular States and Dynamics

The therapeutic landscape for ALI has shifted from broad immunosuppression toward precision immunotherapy targeting specific cellular states and dynamic transitions [144, 145, 146, 147]. Rather than depleting entire populations, these strategies reprogram cellular phenotypes through highly selective delivery systems [65, 145].

Macrophage‐centered approaches have advanced considerably. Biomimetic nanoplatforms using macrophage membrane coating exploit inflammatory tropism for precise lung delivery [146]. Fucoidan‐based systems activate Nrf2 to inhibit ROS synthesis while scavenging existing ROS [145]. Peimine‐loaded macrophage membrane‐coated nanoparticles promote M2 polarization by downregulating NF‐κB and janus kinase (JAK)/STAT pathways [146]. Apoptotic body‐inspired nanoplatforms replicate “eat‐me” signaling for macrophage targeting, delivering mitochondrial‐targeting nanozymes that restore redox homeostasis [147]. Genetic approaches enable precise metabolic reprogramming; inhalable CRISPR/Cas9 nanoplatforms targeting hexokinase 2 in macrophages reduce glycolysis and inflammation [65].

T cell subset‐specific interventions have emerged. CD8+ T cell‐targeted nanoparticles delivering ferrostatin‐1 inhibit ferroptosis and regulate PI3K/Akt and MAPK pathways [148]. Regulatory T cells are critical for resolution, with leukotriene B4 receptor 1 (BLT1)‐dependent alveolar recruitment essential for recovery [149]. Dendritic cell targeting shows promise; CXCR1 depletion in Ly6C+ cDC2 shifts T cell differentiation toward regulatory T cells (Tregs) and attenuates injury [150]. NET formation represents a key pathogenic mechanism; methyltransferase‐like 3 (METTL3)‐mediated m6A methylation of spleen tyrosine kinase (SYK) mRNA promotes NETosis, and myeloid‐specific METTL3 deletion reduces lung injury [151]. Ring finger protein 128 (RNF128) inhibits neutrophil activation by binding myeloperoxidase and targeting TLR4 for degradation [152].

Extracellular vesicle (EVs)‐based therapies offer cell‐free alternatives. Human bronchial epithelial cell‐derived EVs reduce proinflammatory cytokine secretion via miRNAs and annexin A1 (ANXA1)‐mediated FPR2 signaling [153]. Engineered EVs carrying let‐7a‐5p reduce macrophage infiltration and collagen deposition [154]. Adipose‐derived mesenchymal stem cell (MSC) exosomes transfer mitochondrial components to alveolar macrophages, restoring mitochondrial function [155]. Multitarget approaches address ALI complexity through inhalable nanoplatforms combining DNase I and sivelestat [156], redox‐reprogramming strategies [157], and selenium‐based nanoparticles [158].

Natural products continue to yield precision immunomodulators. Norwogonin inhibits proto‐oncogene tyrosine‐protein kinase Src (Src)/AKT1/NF‐κB signaling through direct Src, AKT1, and cyclooxygenase‐2 (COX‐2) targeting [159]. Sanzi Yangqin Decoction regulates TLR2/NF‐κB/NLRP3 signaling [77]. Luteolin acts as a natural BTK and fmss‐like tyrosine kinase 3 (FLT3) inhibitor [160]. Inosine directly binds TLR4, promoting M2 polarization [161]. Shikonin activates mitochondrial mitochondrial calcium uniporter (MCU)/mCa2+ signaling, shifting macrophages from glycolysis to oxidative phosphorylation [162]. Strictosamide regulates Th17/Treg balance via STAT3/STAT5 [163]. Chebulinic acid inhibits IDO1–kynurenine (Kyn) axis activation [64]. Houttuynia cordata‐derived acetate, produced through Phocaeicola vulgatus interaction, activates GPR43 and inhibits JAK2/STAT3, restoring Th17/Treg balance [59]. Bacillus safensis‐derived esculin inhibits TLR2–MyD88–NF‐κB and activates Nrf2–antioxidant response element (ARE), decreasing M1 and increasing M2 polarization [60].

Future directions include integrating single‐cell multiomics to identify novel disease‐associated cellular states, developing inhalable formulations for direct pulmonary delivery, and engineering “smart” nanoplatforms responsive to the inflammatory microenvironment [22, 42, 164]. These approaches collectively move ALI therapy toward true precision medicine, where interventions target specific cellular states and dynamics tailored to individual patients.

### 4.3 Advanced Therapeutic Platforms: Nanomedicine and Endogenous System Engineering

**FIGURE 6:**
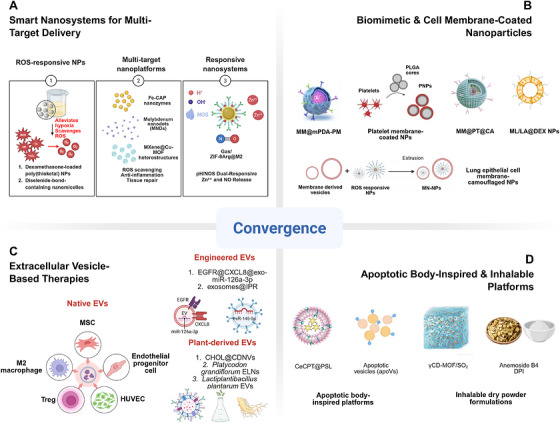
Advanced therapeutic platforms: smart nanomaterials and endogenous system engineering. Overview of next‐generation ALI therapeutics integrating smart nanomaterials with endogenous systems. (A) Smart nanosystems: ROS‐responsive nanoparticles (dexamethasone‐poly(thioketal), diselenide nanomicelles); multitarget nanoplatforms (Fe–CAP nanozymes, molybdenum nanodots, MXene@Cu–MOF, gastrodin‐loaded ZIF–8Arg@M2); responsive nanosystems (acid/NOS dual‐responsive ZIF‐8). (B) Biomimetic nanoparticles: macrophage, platelet, lung epithelial, M2 macrophage, and myeloid cell membrane‐coated platforms. (C) Extracellular vesicle‐based therapies: native EVs (MSC, endothelial progenitor, HUVEC, Treg, M2 macrophage); engineered EVs (EGFR‐conjugated, magnetically navigated exosomes); plant‐derived EVs (Platycodon grandiflorum ELNs, Lactiplantibacillus EVs). (D) Apoptotic body‐inspired platforms (CeCPT@PSL, MSC‐apoVs) and inhalable platforms (γCD–MOF/SO_2_, anemoside B4 DPI). Figure created with BioRender (BioRender.com).

Conventional pharmacotherapy for ALI is hampered by poor bioavailability, off‐target toxicity, and inability to address complex pathological networks [165, 166, 167, 168]. These limitations have spurred development of advanced platforms integrating smart nanomaterials with endogenous biological systems [147, 169, 170, 171, 172]. The diverse array of next‐generation therapeutic platforms is comprehensively overviewed in Figure 6 and cataloged in Table 6.

**TABLE 6:** Emerging therapeutic platforms for ALI: from network pharmacology to endogenous system engineering.

Therapeutic strategy	Platform/approach	Key mechanism of action	Targeting/delivery	Specific examples (with references)
Network pharmacology	Multilevel hub engagement	Ligand clearance, signal transducer inhibition, upstream stabilization, combined hub inhibition	Systemic or local; pulmonary delivery	Ligand clearance: cfDNA‐scavenging cationic nanoparticles [31] Signal transducer inhibition: Inhalable CoAl–LDH nanosheets delivering STING inhibitor C176 [35] Upstream stabilization: TAT–PBX1 (mitochondrial biogenesis) [32]; glabridin (ME1 stabilization) [33] Combined hub inhibition: Shuangdan Jiedu Decoction (cGAS–STING + NLRP3) [123]; Lonicerae–Forsythiae pair [132]
Precision immunotherapy: macrophage‐centered	Biomimetic and cell membrane‐coated NPs	Inflammatory tropism, immune evasion, M2 repolarization	Lung‐homing; intravenous or inhaled	Fucoidan‐based systems (MF@CB) activating Nrf2 [138]; macrophage membrane‐coated Peimine NPs (MM@mPDA–PM) [139]; apoptotic body‐inspired nanoplatforms (CeCPT@PSL) delivering mitochondrial‐targeting nanozymes [140]
	Genetic reprogramming of macrophages	CRISPR/Cas9‐mediated metabolic modulation	Lung‐localized (inhalable)	HK2‐targeting CRISPR/Cas9 nanoplatforms (CSN–mCas9/gHK2) reducing glycolysis and inflammation [58]
Precision immunotherapy: other immune cells	T Cell‐targeted NPs	Inhibition of ferroptosis in CD8+ T cells; Treg recruitment modulation	Intravenous; targeted delivery	CD8+ T cell‐targeted nanoparticles (CD8@PD‐1‐CTL‐M@PLGA/Fer‐1) delivering ferrostatin‐1 [141]
	Dendritic cell modulation	CXCR1 depletion shifts Th17/Treg balance	Systemic (genetic or pharmacological)	CXCR1 depletion in Ly6C+ cDC2 attenuates injury via MEK1/ERK/NF‐κB modulation [143]
	Neutrophil‐targeted therapies	Inhibition of NETosis; targeting METTL3–SYK or RNF128	Systemic or local	METTL3 inhibitors [144]; RNF128 overexpression [145]; inhalable nanoplatforms combining DNase I and sivelestat [149]
Extracellular vesicle (EV)‐based therapies	MSC‐derived EVs	Mitochondrial transfer; miRNA delivery (miR‐125b‐5p, miR‐145‐5p) restoring mitochondrial function, promoting M2 polarization	Inhalation (outperforms IV) [187]; intravenous	MSC‐EVs transferring mitochondrial components [148]; BMSC‐derived exosomal miR‐125b‐5p targeting STAT3 [191]; adipose‐derived MSC exosomes delivering miR‐145‐5p targeting KLF5 [192]
	Endothelial/epithelial cell‐derived EVs	miRNA delivery (miR‐218, miR‐520d‐3p); ANXA1‐mediated FPR2 signaling	Intravenous; intratracheal	HUVEC‐derived EVs delivering miR‐520d‐3p to restore mitochondrial dynamics [194]; endothelial progenitor cell‐derived EVs carrying miR‐218 targeting HMGA1 [193]; HBEC‐EVs with ANXA1 cargo [146]
	Regulatory immune cell‐derived EVs	Immunosuppression; M1‐to‐M2 transition	Nebulization; intravenous	M2 macrophage‐derived EVs modulating P62‐Keap1‐Nrf2 pathway [196]; Treg‐derived EVs suppressing effector T cell responses [195]
	Engineered EVs	Enhanced targeting and potency via surface modification and cargo loading	Cell‐specific targeting; magnetically navigated	CXCL8‐overexpressing macrophage‐derived exosomes loaded with miR‐126a‐3p modified with EGFR antibodies (EGFR@CXCL8@exo‐miR‐126a‐3p) [190]; Magnetically navigated exosomes from iron oxide nanoparticle‐preconditioned BMSCs (exosomes@IPR) [166, 192]
	Plant‐derived EVs	Anti‐inflammatory, metabolic modulation, M2 repolarization; ferroptosis inhibition	Oral; systemic	Platycodon grandiflorum ELNs regulating glycolysis/lipid metabolism [119]; CHOL@CDNVs enhancing macrophage uptake [188]; Lactiplantibacillus plantarum EVs (LpEVs) delivering cbn‐let‐7 targeting Acsl4, activating NRF2/HO‐1/GPX4 [189]
Apoptotic body‐inspired platforms	Phosphatidylserine‐containing liposomal shells (CeCPT@PSL)	Replicate “eat‐me” signaling for macrophage targeting; deliver mitochondrial‐targeting nanozymes for precision mitochondrial restitution	Inhalation	CeCPT@PSL enhancing macrophage repolarization, resolving mitochondrial impairment [140]; apoptotic vesicles (apoVs) from MSCs inhibiting platelet activation, NETosis via CD73 [197]
Smart nanosystems (ROS‐responsive)	Poly(thioketal) nanoparticles; diselenide‐bond‐containing nanomicelles	Triggered drug release under high ROS conditions in inflammatory microenvironment	Pulmonary (inhaled); systemic	Dexamethasone‐loaded poly(thioketal) NPs (PTKNPs@Dex) [169, 170]; diselenide‐bond‐containing nanomicelles enabling precise release at injury sites [168, 171]
Multitarget nanoplatforms	Iron–capsaicin nanozymes (Fe–CAP NPs); molybdenum nanodots (MNDs); MXene@Cu–MOF heterostructures	Simultaneous ROS scavenging, anti‐inflammation, tissue repair; suppression of pyroptotic pathways	Intravenous; inhaled	Fe–CAP NPs combining antioxidant properties with catalytic activity [172]; MNDs suppressing NLRP3‐dependent pyroptotic pathways [35, 173]; MXene@Cu–MOF heterostructures enabling staged action [184]; gastrodin‐loaded ZIF‐8Arg@M2 NPs reducing apoptosis from 63.6% to 2.9% [175]
Biomimetic and cell membrane‐coated NPs	Macrophage, platelet, lung epithelial cell membrane coatings	Enhanced targeting, immune evasion, self‐reinforcing targeting	Lung‐homing; intravenous	Macrophage membrane‐coated polydopamine NPs (MM@mPDA–PM) [139]; Platelet membrane‐coated NPs inhibiting platelet activation, NETosis via CD62P blockade [179]; Lung epithelial cell membrane‐camouflaged ROS‐activatable berberine NPs [171]; M2 macrophage membrane‐coated nanomicelles (MM@PT@CA) restoring cell viability from 48.7 to 93.9% [181]; myeloid cell membrane–chimeric liposomes (ML/LA@DEX NPs) achieving self‐reinforcing targeting [182]
Inhalable dry powder formulations	Food‐grade metal–organic frameworks (γCD‐MOF); anemoside B4 dry powder inhalers	Targeted pulmonary delivery; gas messenger release (SO_2_); enhanced bioavailability	Inhalation	γCD–MOF loaded with SO_2_ achieving 40% fine particle fraction, 92% release within 5 min, regulating P38/NF‐κB pathways [185]; anemoside B4 DPI achieving 18.6% absolute bioavailability (74‐fold increase over oral) with efficacy in high‐altitude ALI [186]

This comprehensive table catalogs the emerging therapeutic platforms for ALI, organized by therapeutic strategy. For each platform, the key mechanism of action, targeting/delivery route, and specific examples with supporting references are provided. The table illustrates the paradigm shift from conventional pharmacotherapy toward network‐informed, precision‐engineered approaches that integrate smart nanomaterials with endogenous biological systems. These strategies encompass network pharmacology, precision immunotherapy targeting specific cellular states, EV‐based therapies, smart nanosystems, biomimetic platforms, and inhalable formulations, collectively representing a new therapeutic armamentarium for ALI.

#### 4.3.1 Smart Nanosystems for Multitarget Delivery

ROS‐responsive nanoparticles leverage elevated reactive oxygen species characteristic of ALI for triggered drug release [173, 174, 175]. Dexamethasone‐loaded poly(thioketal) nanoparticles accumulate in inflamed pulmonary tissues and release payload specifically under high ROS conditions [176, 177]. Diselenide‐bond‐containing nanomicelles enable precise release at injury sites [175, 178]. Multitarget nanoplatforms address multiple pathological drivers simultaneously: iron–capsaicin nanozymes combine antioxidant properties with catalytic activity [179]; molybdenum nanodots suppress NLRP3‐dependent pyroptotic pathways [42, 180]; selenium‐based nanoparticles protect against oxidative injury by maintaining mitochondrial function [181]. Acid and NOS dual‐responsive zeolitic imidazolate framework‐8 (ZIF‐8) nanoparticles incorporating l‐arginine enable controlled Zn^2+^ and nitric oxide release, improving microcirculation [182]. Lung‐targeting NIR‐II ratiometric fluorescent nanoprobes enable real‐time monitoring via hypochlorous acid imaging [183, 184].

Biomimetic and cell membrane‐coated nanoparticles have emerged for enhanced targeting and immune evasion [145, 146, 185, 186, 187]. Macrophage membrane‐coated polydopamine nanoparticles loaded with Peimine reduce neutrophil infiltration and NET formation while promoting M2 polarization [146]. Platelet membrane‐coated nanoparticles inhibit platelet activation and NETosis through CD62P blockade [186]. Lung epithelial cell membrane‐camouflaged ROS‐activatable berberine nanoparticles achieve targeted delivery to injured alveolar epithelium [178]. M2 macrophage membrane‐coated nanomicelles encapsulating carnosic acid restore cell viability under oxidative stress [188]. Myeloid cell membrane‐chimeric liposomes achieve self‐reinforcing targeting by activating CD11b on myeloid membranes [189]. Cascade‐responsive systems represent next‐generation smart nanomedicines [189, 190, 191]. Food‐grade metal‐organic frameworks loaded with sulfur dioxide as dry powder inhalers achieve targeted pulmonary delivery [192]. Anemoside B4 dry powder inhalers achieve 74‐fold increased bioavailability with efficacy in high‐altitude ALI [193].

#### 4.3.2 Harnessing Endogenous Systems: Extracellular Vesicles and Regulatory Immune Cells

Parallel strategies harness endogenous systems, particularly extracellular vesicles and regulatory immune cells [155, 194]. EVs offer inherent biocompatibility, low immunogenicity, and capacity to deliver complex bioactive cargo [195, 196, 197]. MSC‐derived EVs show particular promise; inhalation outperforms intravenous administration in reducing proinflammatory cytokines and promoting M2 polarization [194]. Mechanistically, MSC‐EVs transfer mitochondrial components to alveolar macrophages, restoring mitochondrial function [155]. Bone marrow mesenchymal stem cell (BMSC)‐derived exosomal miR‐125b‐5p targets STAT3 to suppress macrophage pyroptosis [198]. Adipose‐derived MSC exosomes deliver miR‐145‐5p to target KLF5, inhibiting NF‐κB activation [199]. Endothelial progenitor cell‐derived exosomes carrying miR‐218 mitigate sepsis‐induced ALI by targeting high mobility group AT‐hook 1 (HMGA1) [200]. Human umbilical vein endothelial cells (HUVEC)‐derived exosomes deliver miR‐520d‐3p to restore mitochondrial dynamics [201]. Treg‐derived EVs suppress effector T cell responses [202, 203]. M2 macrophage‐derived EVs administered via nebulization promote M1‐to‐M2 transition [203]. Plant‐derived EVs offer scalability and inherent anti‐inflammatory properties [195, 196]. Engineered EVs enhance targeting and potency through surface modifications [148, 173, 197, 199]. Apoptotic body‐inspired nanoplatforms converge synthetic and endogenous approaches [147, 204].

### 4.4 Translational Hurdles and the Evolving Clinical Trial Landscape

The journey from promising preclinical network‐based therapeutics to clinical reality in a heterogeneous syndrome like ARDS is fraught with challenges. Past efforts targeting single pathways, while scientifically sound, have often yielded disappointing results in clinical trials, providing critical lessons for future drug development. For instance, while beta‐2 adrenergic receptor agonists showed promise in preclinical models by increasing cyclic adenosine monophosphate (cAMP) and protecting alveolar epithelium [205], large clinical trials in ARDS patients failed to demonstrate a mortality benefit, underscoring the critical importance of patient selection and timing of intervention [205]. Similarly, the neutrophil elastase inhibitor sivelestat, despite being approved for ALI/ARDS in Japan and South Korea, has shown conflicting results in broader populations, highlighting the need to identify responsive subphenotypes, such as hyperinflammatory patients, who may derive the greatest benefit [206, 207, 208]. These historical experiences have paved the way for a new generation of clinical trials exploring multitargeted or repurposed agents.

Dipyridamole (DIPY) has emerged as a promising repurposed candidate with a network‐based mechanism. A screening of 259 United States Food and Drug Administration‐approved drugs identified DIPY as a potent ferroptosis inhibitor in pulmonary epithelial and endothelial cells, acting through superoxide dismutase 1 (SOD1) activation to suppress the CAMP responsive element binding protein 1 (CREB1)/heme oxygenase 1 (HMOX1) pathway. A proof‐of‐concept clinical trial subsequently demonstrated improved outcomes with DIPY adjunctive therapy in ARDS patients, providing early clinical validation for therapies targeting ferroptosis, a key cell death pathway discussed earlier [209].

The JAK–STAT pathway, central to cytokine signaling, has also been therapeutically targeted. Ruxolitinib, a JAK1/JAK2 inhibitor, was evaluated in mechanically ventilated COVID‐19 ARDS patients. The Phase 3 RUXCOVID‐DEVENT trial (NCT04377620) reported a 28‐day mortality of 51–53% with ruxolitinib versus 70% with placebo; however, this difference was not statistically significant, partly due to early termination of the study [210]. A smaller Phase II study (NCT04359290) nonetheless demonstrated the feasibility of this approach in 16 mechanically ventilated patients, with 13 surviving the first 28 days [211].

Cell‐based therapies, particularly those using MSCs, have also progressed to later‐stage trials, with mixed results that underscore the importance of biological heterogeneity. The Phase 2b STAT trial (NCT03818854) randomized 120 ventilated ARDS patients to intravenous MSCs or placebo. While no difference in the primary outcome (oxygenation index) or mortality was observed in the overall cohort, biomarker analyses identified patient subgroups with differential treatment responses, suggesting that MSC therapy may be beneficial only in specific endotypes [212]. Despite this, a meta‐analysis of 48 studies including 1773 patients demonstrated a significant mortality reduction within 1 month (RR = 0.74, 95% CI 0.63–0.87), particularly with high‐dose MSCs, and MSC‐derived extracellular vesicles showed promising efficacy in COVID‐19‐associated ARDS (RR = 0.63, 95% CI 0.46‐0.86) [213, 214].

### 4.5 The Road to Clinical Translation: Challenges and Opportunities

The systems‐level understanding of ALI as a network disorder necessitates a corresponding evolution in therapeutic strategy. Rather than targeting isolated effectors—an approach that has yielded disappointing results in clinical trials of agents such as β_2_‐agonists and neutrophil elastase inhibitors [205, 206, 207, 208]—next‐generation therapies are designed to disrupt network hubs, reprogram cellular states, and harness endogenous reparative mechanisms. Network pharmacology offers multilevel hub engagement targeting cGAS–STING, NLRP3, and PANoptosis platforms. Precision immunotherapy enables cell‐type‐specific modulation through biomimetic nanoparticles, genetic reprogramming, and engineered extracellular vesicles. Advanced nanomedicine platforms integrate smart nanomaterials with endogenous systems for targeted, multitarget delivery.

However, clinical translation faces substantial hurdles that mirror the lessons learned from past failures. Patient stratification remains paramount; the identification of hyperinflammatory (C1) and hypoinflammatory (C2) endotypes has demonstrated that molecularly defined subgroups exhibit differential treatment responses to both standard interventions (e.g., positive end‐expiratory pressure [PEEP], glucocorticoids) and emerging therapies [116, 120]. The conflicting results from trials of sivelestat underscore this reality—benefits may be confined to specific subphenotypes, such as hyperinflammatory patients with excessive neutrophil elastase activity. Inconsistent trial results with agents like ruxolitinib and MSCs further highlight the inadequacy of traditional preclinical models and the necessity for innovative trial designs that accommodate biological heterogeneity. Finally, scalability and guanosine monophosphate (GMP) compliance for cell‐based and nanomedicine platforms require standardized, reproducible protocols before widespread adoption becomes feasible. Ongoing trials provide critical insights, but larger, endotype‐guided studies are urgently needed to establish efficacy in molecularly defined patient populations.

## 5 Conclusion and Future Perspectives: Toward Network‐Based Therapeutics

The study of ALI has reached a transformative juncture. The limitations of reductionist models—evidenced by the repeated failure of single‐target interventions in clinical trials—are now unmistakable, and a new framework centered on network pathophysiology is coalescing. Our analysis demonstrates that ALI represents a systemic failure of homeostasis involving interdependent molecular circuits: innate immune surveillance (exemplified by the cGAS–STING pathway), immunometabolic reprogramming (including succinylation and lactylation modifications), and organelle‐driven cell death (ferroptosis, PANoptosis). These circuits communicate across multiple organs through neural, humoral, and microbial signals, producing the profound clinical heterogeneity that has long frustrated therapeutic development. This reconceptualization provides a coherent explanation for this heterogeneity and offers a rational basis for therapeutic innovation, as outlined in the roadmap presented in Figure 5.

The convergence of single‐cell technologies, multiomics platforms, and computational modeling is rapidly transforming our ability to dissect this complexity. Three critical domains require focused efforts to bridge the gap between preclinical promise and clinical reality.

First, achieving diagnostic precision through integrated multiomics is essential for patient stratification. The reproducible identification of inflammatory endotypes—hyperinflammatory (C1), hypoinflammatory (C2), and intermediate (C3)—with differential treatment responses to glucocorticoids and PEEP demonstrates that molecular stratification is clinically actionable [116, 120]. Point‐of‐care assays for real‐time endotype assignment must be developed to enable precision medicine in critical care settings, potentially rescuing therapies like sivelestat that may benefit only a subset of patients.

Second, therapeutic development must pivot from targeting isolated effectors to disrupting network hubs and modules. The cGAS–STING pathway, NLRP3 inflammasome, and PANoptosis platform exemplify high‐priority nodes where multilevel intervention strategies—from ligand clearance to signal transducer inhibition to upstream organelle stabilization—can achieve durable network reconfiguration. Rationally designed poly‐pharmacology, exemplified by multiherb formulations and multitarget nanomedicines, offers a means to engage multiple vulnerable nodes simultaneously, preventing adaptive resistance and amplifying efficacy.

Third, rigorous attention to translational barriers is required to ensure that preclinical promise translates to clinical reality. These barriers include the fidelity of animal models to human ARDS, the immunogenicity and unpredictable pharmacokinetics of novel nanomedicines, and the scalability and Good Manufacturing Practice compliance of cell‐derived therapeutics such as extracellular vesicles. Organ‐on‐chip platforms and artificial intelligence‐driven predictive models can accelerate the screening and optimization of candidate interventions before costly clinical trials. The ongoing trials discussed herein represent important steps toward clinical validation, but larger, endotype‐guided studies are needed to establish efficacy in molecularly defined patient subgroups, learning from the ambiguous results of trials like RUXCOVID–DEVENT and STAT.

Looking forward, the integration of systems biology with advanced therapeutic platforms holds the potential to transform ALI from a supportive care paradigm to one of genuine disease modification. Smart nanomaterials that respond dynamically to the inflammatory microenvironment, engineered EVs that harness endogenous reparative mechanisms, and precision immunotherapies that reprogram specific cellular states collectively represent a new therapeutic armamentarium. The ultimate goal is not merely to manage pulmonary inflammation but to restore the complex biological networks that maintain lung homeostasis—a vision that defines the next era of critical care medicine, where insights from network medicine finally translate into durable clinical benefit for patients with this devastating syndrome.

## Author Contributions

Concept and design: R. Jing and W.J. Liu. Acquisition, analysis, or interpretation of data: Y.L. Hou, S. He, L.L. He, and R. Jing. Drafting of the manuscript: Y.L. Hou, Z.Y. Zeng, and Y. Wang. Critical review of the manuscript for important intellectual content: K. Liu, W. Tang, D.M. Wang, and J. Gui. Figure illustration: Y.L. Hou and R. Jing. Administrative, technical, or material support: K. Liu, W. Tang, D.M. Wang, and J. Gui. Supervision: R. Jing, W.J. Liu, Z.Y. Zeng, and Y. Wang. All authors have read and approved the final manuscript.

## Funding

This study was funded by the National Natural Science Foundation of China (82500127), Guangdong Basic and Applied Basic Research Foundation (2023A1515110149), Shenzhen Science and Technology Program (JCYJ20240813144035045), Key R&D Program of Hunan Provincial Natural Science Funds (No. 2024JK2131 to W.L.), and Hunan Clinical Research Center for Acute and Chronic Pain (No. 2023SK4014 to W.L.).

## Ethics Statement

This article is a literature review focusing on the research progress of acute lung injury (ALI) and acute respiratory distress syndrome (ARDS). All analyses in this study were performed based on previously published and publicly available literature data. No human participants, human tissues, or animal experiments were involved in this study. Therefore, ethical approval and informed consent were not required.

## Conflicts of Interest

The authors declare no conflicts of interest.

## Data Availability

The authors have nothing to report.
